# Cooperativity in Binding Processes: New Insights from Phenomenological Modeling

**DOI:** 10.1371/journal.pone.0146043

**Published:** 2015-12-30

**Authors:** Diego I. Cattoni, Osvaldo Chara, Sergio B. Kaufman, F. Luis González Flecha

**Affiliations:** 1 Laboratorio de Biofísica Molecular, Instituto de Química y Fisicoquímica Biológicas, Universidad de Buenos Aires – CONICET, Buenos Aires, Argentina; 2 Centre de Biochimie Structurale, Université de Montpellier 1 and 2, Montpellier, France; 3 Institute of Physics of Liquids and Biological Systems (IFLYSIB), University of La Plata - CONICET, La Plata, Argentina; 4 Center for Information Services and High Performance Computing, Technische Universität Dresden, Dresden, Germany; University of Copenhagen, DENMARK

## Abstract

Cooperative binding is one of the most interesting and not fully understood phenomena involved in control and regulation of biological processes. Here we analyze the simplest phenomenological model that can account for cooperativity (i.e. ligand binding to a macromolecule with two binding sites) by generating equilibrium binding isotherms from deterministically simulated binding time courses. We show that the Hill coefficients determined for cooperative binding, provide a good measure of the Gibbs free energy of interaction among binding sites, and that their values are independent of the free energy of association for empty sites. We also conclude that although negative cooperativity and different classes of binding sites cannot be distinguished at equilibrium, they can be kinetically differentiated. This feature highlights the usefulness of pre-equilibrium time-resolved strategies to explore binding models as a key complement of equilibrium experiments. Furthermore, our analysis shows that under conditions of strong negative cooperativity, the existence of some binding sites can be overlooked, and experiments at very high ligand concentrations can be a valuable tool to unmask such sites.

## Introduction

Specific recognition and interaction between macromolecules and ligands determines the fate of most cellular processes and thus the behavior, response and regulation of essential functions in all living organisms [1,2]. Regulation of gene expression [3], enzyme activities [4,5], protein stabilization [6,7], cell membrane electrochemical potential [8], oxygen transport [9] and neural proliferation [10] are only a few examples of the great diversity of phenomena that occur in biological systems as a consequence of intermolecular interactions. Despite its key role, the underlying relationships and mechanisms are still a matter of debate.

Cooperative binding represents perhaps one of the most interesting, and not fully understood, types of molecular interactions observed in nature [11]. For macromolecules having two or more binding sites, cooperativity is characterized by a change of the intrinsic (site specific) equilibrium binding constant as a function of the reaction progress (i.e. the affinity of a given binding site for a ligand will be affected by the occupancy of other sites by the same or different ligands). The first modeling approaches describing cooperative binding were proposed by A.V. Hill at the beginning of the twentieth century by analyzing the binding of oxygen to human hemoglobin [12,13]. At that time, hemoglobin was thought to be a monomeric molecule containing one atom of iron [14]. To conciliate this data with the sigmoidal shape of the oxygen binding curve, Hill proposed that these monomers aggregate in groups of *n* units, and that this ‘aggregate’ bound *n* molecules of oxygen simultaneously [14]. From this model a mathematical expression (Eq 1) was derived [13], where the fractional saturation of hemoglobin by O_2_ (θ) is expressed as a function of the partial pressure of O_2_ or, for the general case, the free ligand concentration [*L*]. This equation includes two parameters: an association constant (*K*) and an exponent affecting the ligand concentration today denoted as the Hill coefficient (*n*_H_).

$$\theta =\frac{K\cdot {\left[L\right]}_{}{}^{n_{\text{H}}}}{1^{}+K\cdot {\left[L\right]}_{}{}^{n_{\text{H}}}}$$ (1)

Later on, Adair et al. reevaluated the molecular weight of hemoglobin demonstrating that four iron ions are present per molecule [15]. Thus, the authors took into account the existence of four oxygen-binding sites, and by applying the mass action law to the corresponding association equilibriums obtained an equation that describes the binding curve containing four equilibrium constants, one for each sequential binding step [15]. By comparing this expression with that proposed by Hill, Adair concluded that the Hill equation would represent a limit situation where the partially saturated states either do not exist or do not contribute to the binding curve; this extreme condition is now denoted as infinite interaction.

Despite the fact that these two hypotheses (hemoglobin aggregation and infinite interaction) are perhaps unrealistic, Eq 1 is still nowadays the most used mathematical expression to describe cooperative binding in the scientific literature. In this scenario, it is commonly accepted that the Hill coefficient provides a criterion to determine the type of interaction between binding sites in a macromolecule. When the value of the Hill coefficient is 1, the Hill equation becomes a rectangular hyperbola indicating that there is no interaction among binding sites (referred hereafter as identical and independent sites). If *n*_H_ takes values higher than 1, it is said that the system shows positive cooperativity; this could be the result of an increase in the affinity of a binding site due to the previous binding of a ligand to another site. Instead, *n*_H_ values lower than 1 would indicate negative cooperativity (also called antagonism) and, in this case, the binding of the first ligand molecule diminishes the probability of binding for a second molecule. However, the condition *n*_H_ < 1 while being necessary, is not sufficient to probe the existence of negative cooperativity, since macromolecules with multiple binding sites and different ligand affinities will also depict *n*_H_ values lower than the unity [16]. Although there are few well-documented cases of negative cooperativity (see Ruzicka & Frey [17], Abeliovich [18] and references therein), its relevance cannot be underestimated. Indeed, Koshland and Hamadani suggested that both, positive and negative cooperativity, are part of a phenomenon of universal importance in biological systems, and have about equal evolutionary relevance [19].

**Fig 1:**
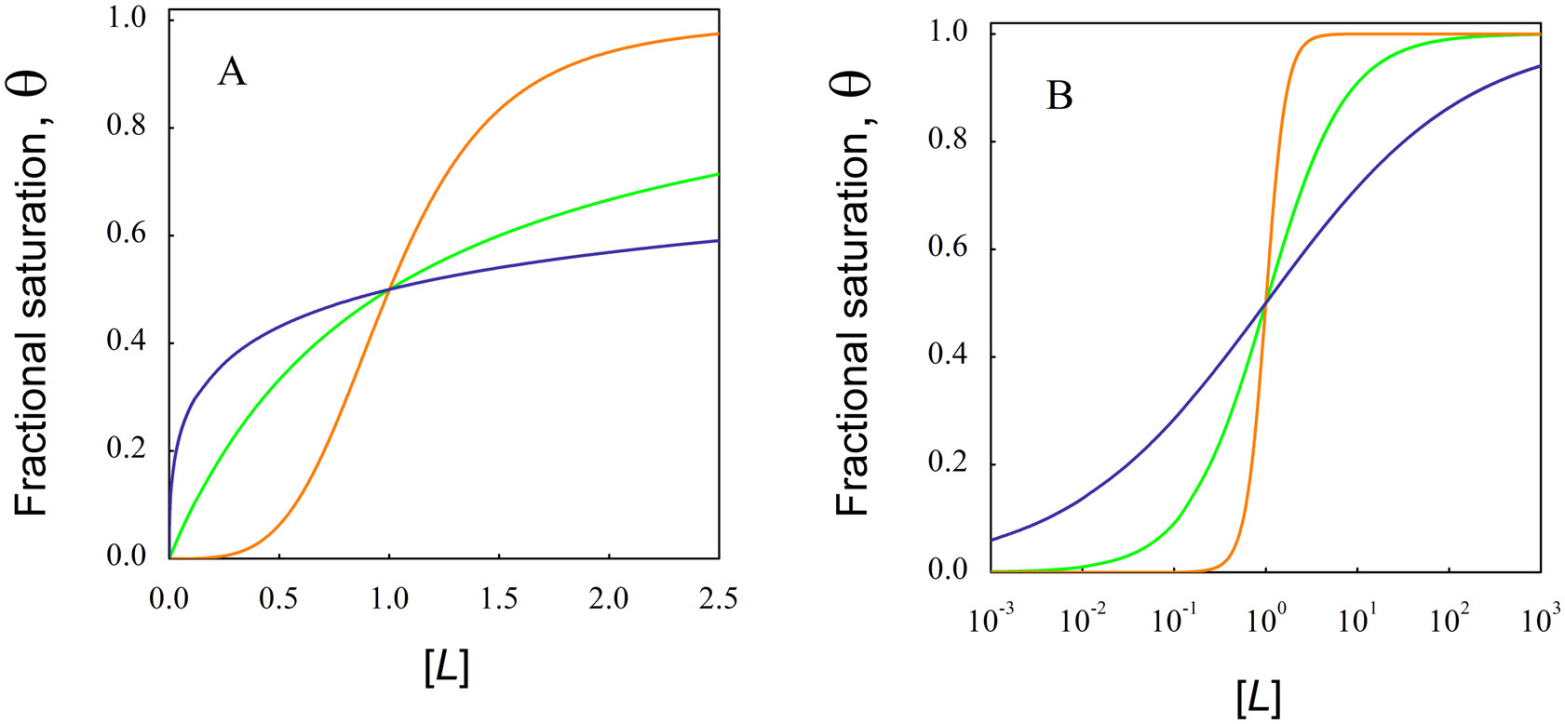
Saturation curves for the three characteristic types of interactions between sites. The fractional saturation of a macromolecule by a ligand was simulated using Eq 1 for identical and independent sites (green line), positive cooperativity (orange line) and negative cooperativity (blue line). Panel A shows a detail of the curves at low ligand concentrations, whereas Panel B includes saturation values for a wide range of ligand concentration in a logarithmic scale

Fig 1 exemplifies the three types of binding interactions between sites, showing the fractional saturation of binding sites in a macromolecule as a function of the free ligand concentration.

It can be observed in Fig 1, the hyperbolic shape of the saturation curve, characteristic of independent sites without interactions (green line in panel A), and the deviations produced by positive (orange line) and negative (blue line) cooperativity. It is worth noting that the sigmoidal shape of the curve is distinctive for positive cooperativity in the direct representation (Panel A). When the curves are represented as a function of the logarithm of ligand concentration (Panel B), an apparent sigmoidicity appears for all types of binding. An useful criteria to analyze binding curves in the logarithmic scale is the so-called “characteristic free-ligand span”, defined as the ratio between the free-ligand concentration corresponding to fractional saturations between 0.1 and 0.9 [20]. This span provides a clear characterization of both the quality of the binding isotherm and the underlying complexity of the binding mechanism [1]. For a single class of binding sites this span equals 81 (1.908 units in the logarithmic scale) and this value strongly diverges towards lower or higher values for positive or negative cooperativity respectively. For experimentally obtained data, this span appears to be more sensitive to distinguish small cooperative interactions from the case of independent sites without interactions than the determination of Hill coefficients close to 1 [16,21].

The large majority of experimental binding studies are performed at equilibrium conditions. In this way, the models used to give account of ligand-macromolecule interactions include only the information corresponding to the final thermodynamic state of the system under study. However, the time evolution of a ligand-binding reaction from pre-equilibrium to equilibrium conditions may provide relevant information related to both, the microscopic configuration of the system -which defines the interaction model- and the molecular mechanism of such process.

In this work, we analyze the simplest microscopic binding scheme that can exhibit cooperativity (i.e. a macromolecule with two binding sites for a ligand). Simulations of the resulting phenomenological models allowed to generate equilibrium binding isotherms from the time courses for each binding model. This approach was then used to gain insights on cooperative binding and analyze the dependence between the Hill coefficient and the model parameters. Additionally, the simulated data was thoroughly explored in search of conditions at which different binding models can be distinguished, and possible hidden binding sites on the macromolecule can be unmasked.

## Methods

### Microscopic description of ligand binding to a macromolecule with two binding sites

**Fig 2:**
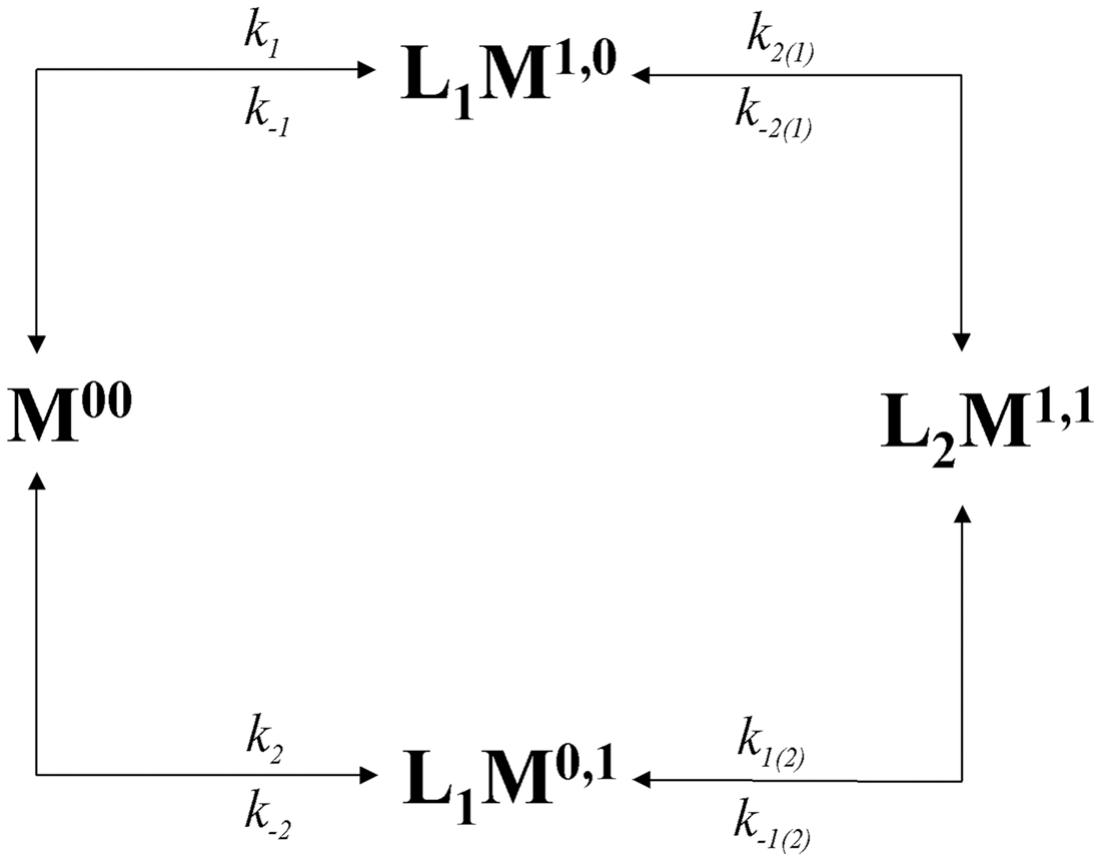
Microscopic reaction scheme for ligand binding to a macromolecule containing two binding sites.

The simplest binding scheme capable of exhibiting cooperativity requires the existence of at least two binding sites. Fig 2 depicts all the possible microscopic states that a single macromolecule (*M*) may acquire as it is occupied sequentially by two ligand molecules (*L*).

The two-digit superscript employed in Fig 2 identifies each single binding site in *M* with a binary notation (0 = empty, 1 = occupied). For example, a macromolecule described by the notation *L*_1_ *M* ^**1,0**^ refers to the microscopic state in which a macromolecule *M* has one ligand (indicated by the subscript 1 of *L*) bound to site 1 while the second site remains empty (indicated by the superscript 1,0 of *M*). The association and dissociation rate coefficients connecting two adjacent microscopic states are identified with a subscript that takes into account the site that is occupied or emptied in each sequential step. Parentheses denote those sites that remain occupied during this ligand-binding step. For example, the notation *k*_-2(1)_ represents the kinetic coefficient for the dissociation of a ligand molecule from site 2, while a second ligand molecule remains bound at site 1. For each step, the ratio between the association and dissociation rate coefficients defines the thermodynamic equilibrium constant for the association of the ligand to that specific site (*K*_oi_), namely the intrinsic (or microscopic) equilibrium constant for ligand association to site *i* [22] (see S1 Appendix for further details).

### Time course simulations

The deterministic kinetic description of the binding models included in the reaction scheme represented in Fig 2 is given by the following set of ordinary differential equations:

$$\frac{d\left[M^{\text{0,0}}\right]}{dt}\;=\;k_{-1}\cdot \left[L_{\text{1}}M^{\text{1,0}}\right]+k_{-2}\cdot \left[L_{\text{1}}M^{\text{0,1}}\right]-(k_{1}+k_{2})\cdot \left[L\right]\cdot \left[M^{\text{0,0}}\right]$$ (2)

$$\frac{d\left[L_{\text{1}}M^{\text{1,0}}\right]}{dt}\;=\;k_{1}\cdot \left[L\right]\cdot \left[M^{\text{0,0}}\right]+k_{-2(1)}\cdot \left[L_{\text{2}}M^{\text{1,1}}\right]-\left(k_{-1}+k_{2(1)}\cdot \left[L\right]\right)\cdot \left[L_{\text{1}}M^{\text{1,0}}\right]$$ (3)

$$\frac{d\left[L_{\text{1}}M^{\text{0,1}}\right]}{dt}\;=\;k_{2}\cdot \left[L\right]\cdot \left[M^{\text{0,0}}\right]+k_{-1(2)}\cdot \left[L_{\text{2}}M^{\text{1,1}}\right]-\left(k_{-2}+k_{1(2)}\cdot \left[L\right]\right)\cdot \left[L_{\text{1}}M^{\text{0,1}}\right]$$ (4)

$$\frac{d\left[L_{\text{2}}M^{\text{1,1}}\right]}{dt}\;=\;\left(k_{1(2)}\left[L_{\text{1}}M^{\text{0,1}}\right]+k_{2(1)}\left[L_{\text{1}}M^{\text{1,0}}\right]\right)\cdot \left[L\right]-\left(k_{-1(2)}+k_{-2(1)}\right)\cdot \left[L_{\text{2}}M^{\text{1,1}}\right]$$ (5)

$$\begin{array}{l} \begin{array}{cc} \frac{d\left[L\right]}{dt}\;= & k_{-1}\cdot \left[L_{\text{1}}M^{\text{1,0}}\right]+k_{-2}\cdot \left[L_{\text{1}}M^{\text{0,1}}\right]+(k_{-2(1)}+k_{-1(2)})\cdot \left[L_{\text{2}}M^{\text{1,1}}\right]-\\ & -\left((k_{1}+k_{2})\cdot \left[M^{\text{0,0}}\right]+k_{2(1)}\cdot \left[L_{\text{1}}M^{\text{1,0}}\right]+k_{1(2)}\cdot \left[L_{\text{1}}M^{\text{0,1}}\right]\right)\cdot \left[L\right] \end{array} \end{array}$$ (6)

Generation of simulated data (i.e. the concentration of each of the species as function of time) requires the numerical solution of these equations. For this purpose an iterative procedure was implemented in Excel spreadsheets by using a 4^th^ order Runge-Kutta algorithm. Simulations were run until not significant changes in the concentration of all species were detected (relative concentration change < 10^−3^%) thus assuming that equilibrium conditions were reached. The binding density 〈*n*〉 is then calculated from the equilibrium concentrations according to Eq 7:

$$\langle n\rangle \equiv \frac{\text{concentration of bound ligand}}{\text{total concentration of macromolecule}}=\frac{\left[L_{1}M\right]+2\cdot \left[L_{2}M\right]}{\left[M\right]+\left[L_{1}M\right]+\left[L_{2}M\right]}=\frac{{\left[L\right]}_{\text{o}}-{\left[L\right]}_{}}{{\left[M\right]}_{\text{o}}}$$ (7)

Where [*L*]_o_ and [*M*]_o_ are the total ligand and the total macromolecule concentrations respectively, [*L*_1_ *M*] is the concentration of the macroscopic state of the macromolecule with one ligand bound (including the microscopic states *M*_1_ *L* ^0,1^ and *M*_1_ *L* ^1,0^), [*L*_*2*_ *M*] is the concentration of the macroscopic state of the macromolecule with two ligands bound and [*L*] the free ligand concentration.

### Hill coefficients

The Hill coefficients were determined by using a refinement of the procedure developed by Jeffries Wyman in the 1960s [23]. Briefly, the simulated binding isotherms were represented in the form of the so-called Wyman’s Hill plot [14]. The Hill coefficient is calculated as the maximum (or minimum) value of the first derivative

$$n_{\text{H}}=\text{T}\left[\frac{\partial \text{ln}\left(\frac{\langle n\rangle }{N-\langle n\rangle }\right)}{\partial \text{ln}\left(\left[L\right]\right)}\right]$$ (8)

where the operator T is either maximum or minimum. The detailed procedure is described in S2 Appendix.

### Association and interaction free energies

Association free energies were calculated from the kinetic coefficients corresponding to each reaction step (*i*) as:

$$\Delta G_{i\;assoc}^{\text{o}}=-R\cdot T\cdot \text{ln}\left(\frac{k_{i}}{k_{-i}}\right)$$ (9)

As defined by Wyman [23], the free energy of interaction between sites will be given by:

$$\Delta G_{\text{int}}^{\text{o}}=\Delta G_{2\;assoc}^{\text{o}}-\Delta G_{1\;assoc}^{\text{o}}=-R\cdot T\cdot \text{ln}\left(\frac{k_{2(1)}}{k_{-2(1)}}\right)+R\cdot T\cdot \text{ln}\left(\frac{k_{1}}{k_{-1}}\right)=-R\cdot T\cdot \text{ln}\omega$$ (10)

where ω represents a cooperativity factor that takes a value equal to one for independent binding sites and any other real positive value for non-independent binding sites (see S1 Appendix for further information).

### Model fitting

A non-linear regression procedure implemented on a Excel spreadsheets was used to fit the binding models to the simulated data [24].

## Results and Discussion

### Equilibrium binding isotherms from simulated binding time courses

Time courses of the reaction between a macromolecule (*M*) containing two binding sites for a ligand (*L*) were simulated by using the microscopic scheme represented in Fig 2. Despite computational simulations deal only with the magnitude of the model variables, the use of physical units can help to connect simulations with experimental results. This is particularly important in those cases involving macromolecule or ligand concentrations, or second order rate coefficients. In this way, total concentration of *M* was kept constant at 1 (μM) for all the simulations, while the concentration of ligand was varied, in regular steps, two orders of magnitude below and above the dissociation constant (assuring in this way, very low and saturating ligand concentrations). For the sake of simplicity, the kinetic association and dissociation coefficients were initially set at *k*_1_ = *k*_1(2)_ = *k*_2_ = *k*_2(1)_ = 1 (μM^-1^s^-1^) and *k*_-1_ = *k*_-1(2)_ = *k*_-2_ = *k*_-2(1)_ = 1 (s^-1^), giving a microscopic association constant *K*_o_ = 1 (μM^-1^). Fig 2 with this set of kinetic coefficients corresponds to the identical and independent sites binding model.

Time course of cooperative ligand binding was simulated by setting the kinetic coefficients for the empty sites at *k*_1_ = *k*_2_ = 1 (μM^-1^s^-1^) and *k*_-1_ = *k*_-2_ = 1 (s^-1^), and the kinetic coefficient for the association of a second ligand molecule when one site is occupied (*k*_1(2)_ = *k*_2(1)_) was varied by multiplying *k*_1_ by a cooperativity factor ω ranged from 0.01 to 100. Note that in these simulations all dissociation rate constants were kept constant at 1 (s^-1^), while the association rate constants range from 0.01 to 100 (μM^-1^s^-1^) covering the range observed for the majority of experimentally characterized second order biochemical reactions [2].

**Fig 3:**
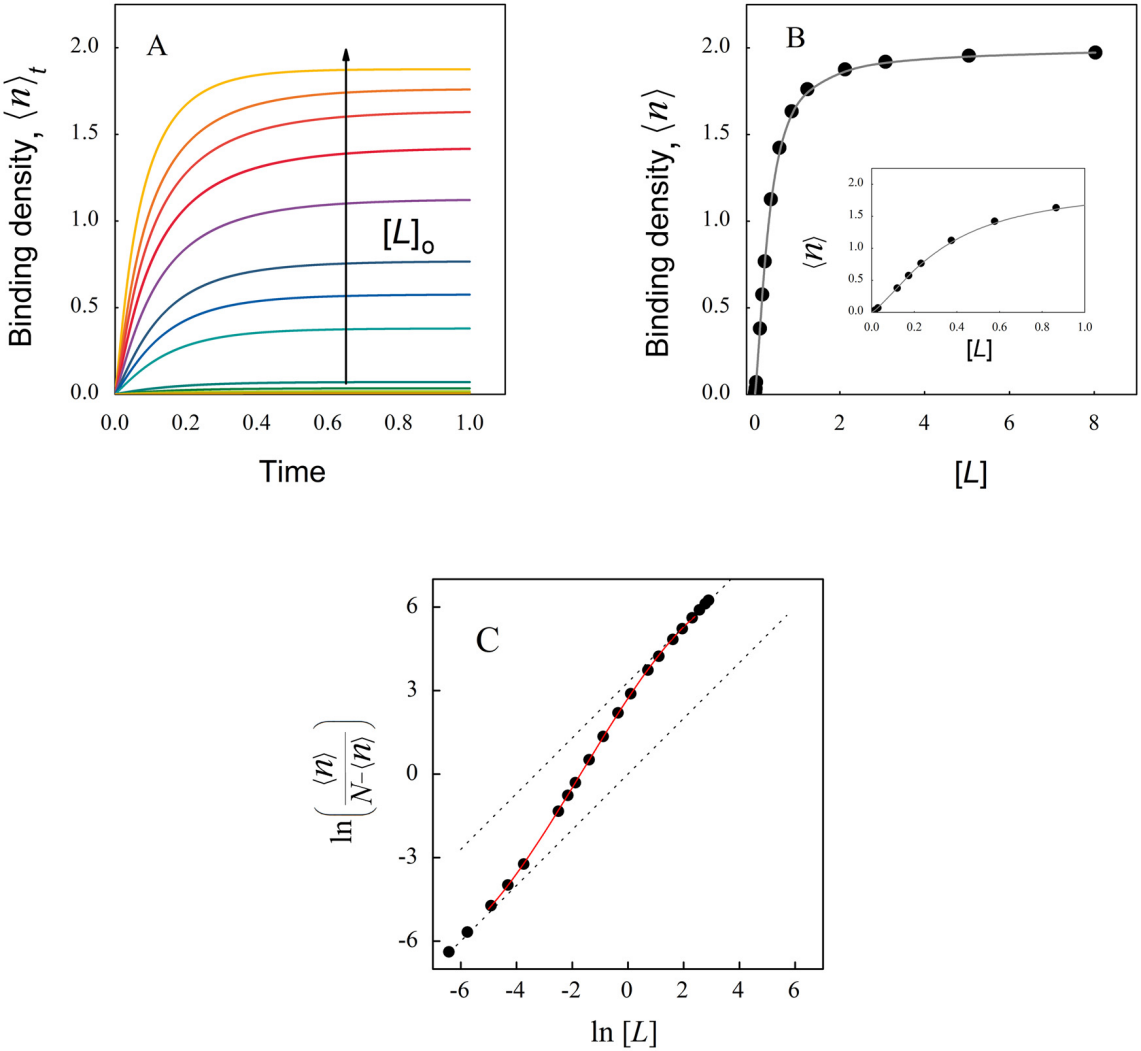
Site occupation for a macromolecule showing positive cooperativity. Eqs 2 to 6 were numerically solved using the kinetic coefficients indicated in the main text for cooperative binding with ω = 10. (A) The average number of occupied sites (〈*n*〉_t_) was calculated using Eq 7 and represented as a function of time (in seconds). Arrow indicates increasing total ligand concentration. (B) The equilibrium values of 〈*n*〉 and [*L*] (μM) were obtained from each time course and plotted as a binding isotherm. (C) Wyman´s Hill plot of data from panel B. At both edges, the calculated data approaches to linear asymptotes with slope unity (dotted lines). The continuous red line represents a third order polynomial fitted to the data in the transition region with the best fitting parameters a_3_ = -0.016; a_2_ = 0.0807 a_1_ = 1.5085 and a_0_ = 2.718. From these values a Hill coefficient of 1.54 was obtained using Eq. B5 (S2 Appendix).

Fig 3A shows the time course for the average number of binding sites of the macromolecule occupied by the ligand *L* (〈*n*〉_t_, denoting the time dependence of the binding density) simulated from the numerical solution of Eqs 2–6 associated to the reaction scheme depicted in Fig 2 with positive cooperativity. When the system reached the equilibrium, the values of 〈*n*〉 and [*L*] were represented as a binding isotherm (Fig 3B).

### Revisiting the Hill coefficient

As it was previously mentioned the Hill equation is commonly used to analyze cooperative binding, although the molecular mechanisms that support this equation does not hold for any real system. In this context, Eq 1 has become a phenomenological model where *K* does not represent a real equilibrium constant. Instead, its root *K* ^1/nH^ represents the reciprocal of the concentration of ligand that produces half saturation, and *n*_H_ is an empirical coefficient giving account of the mismatch respect to the hyperbolic behavior characteristic of binding to identical sites with no interactions.

In the 1960s Jeffries Wyman redefined the Hill coefficient and proposed that it is closely related to the free energy of interaction [23]. Our modeling strategy allowed us to explore this relationship in detail. For this purpose, the equilibrium data shown in Fig 3B was re-plotted in the form of the Wyman’s Hill plot (Fig 3C) assuming that the number of binding sites (*N*) is the asymptotic value of the binding density 〈*n*〉 for each binding isotherm. The Hill coefficient was then calculated in the same way as in a real experimental study.

**Fig 4:**
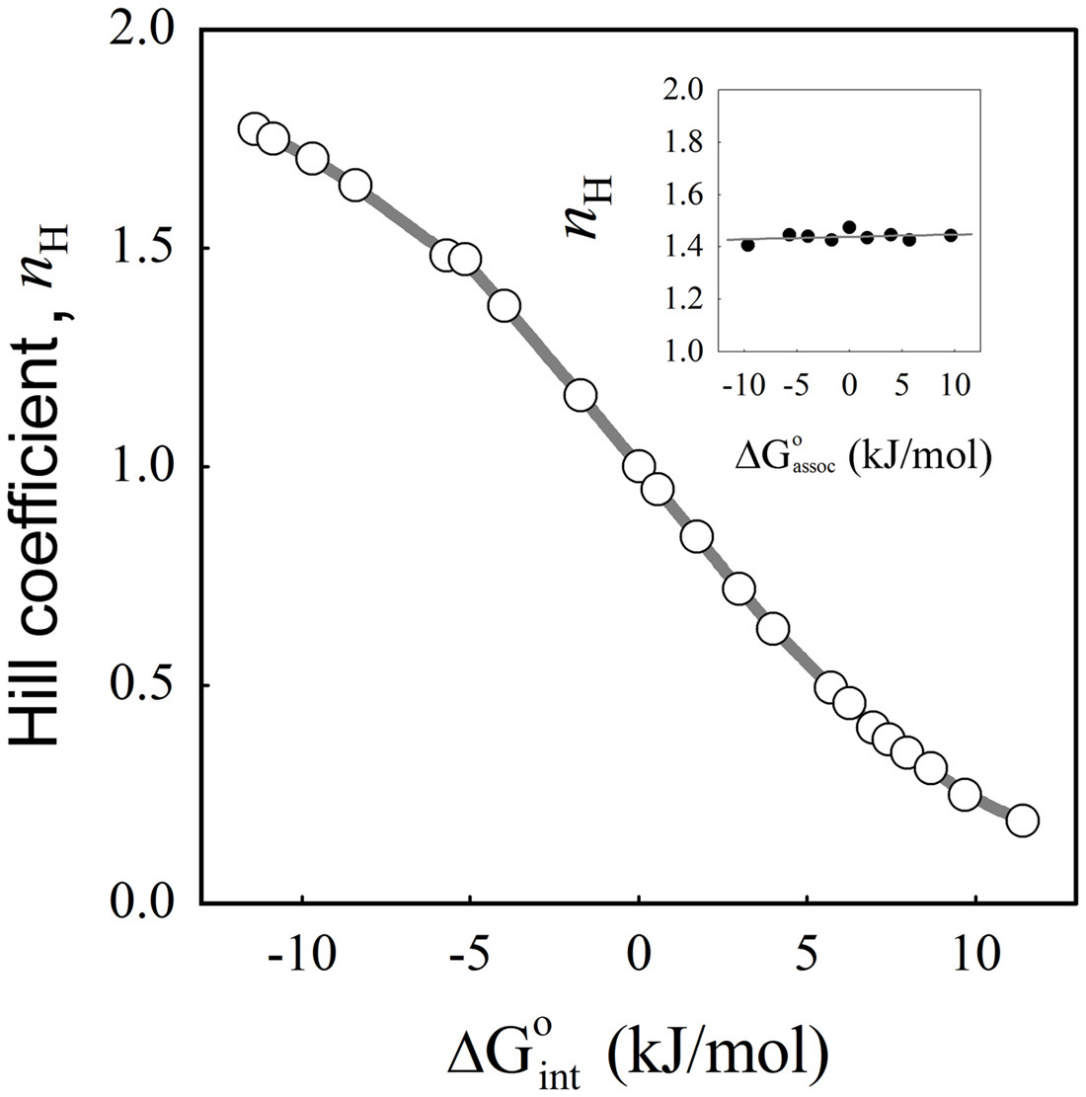
Dependence of the Hill coefficient on the interaction and association free energies. The Hill coefficient was calculated as described in Fig 3 from the binding isotherms obtained for identical sites with *K*_o_ = 1 (μM^-1^) and different values of the cooperativity factor ω (main plot) and for identical sites with *K*_o_ varying from 0.01 to 100 (μM^-1^) and a fixed value of the cooperativity factor ω = 8 (inset). *ΔG* ^o^_int_ (main plot) and *ΔG* ^o^_assoc_ (inset), were calculated using Eqs 10 and 9 respectively. Continuous lines are the graphical representation of polynomial functions fitted to the simulated data.

Fig 4 shows the obtained Hill coefficient values represented as a function of the free energy of interaction calculated for all the ω values using Eq 10. In this plot positive cooperativity corresponds to the region where *n*_H_ > 1 and *ΔG* ^o^_int_ < 0, and negative cooperativity to *n*_H_ < 1 and *ΔG* ^o^_int_ > 0. As it can be observed, the Hill coefficient is a monotonic function of *ΔG* ^o^_int_ in good agreement with the Wyman’s hypothesis. But, is the Hill coefficient a good measure of the interaction energy? Answering this question requires determining whether the Hill coefficient correlates with the intrinsic association constant, independently of the cooperativity factor used to generate the simulated data. Thus, we repeated the previously described procedure but exploring simulated data with intrinsic affinities for empty sites (*K*_o_) ranging from 0.01 to 100 (μM^-1^), and by fixing the value of the cooperativity factor. The inset of Fig 4 shows the obtained values of *n*_H_ represented as a function of the association free energy (*ΔG* ^o^_assoc_) calculated for each condition using Eq 9. Our results show that there is no correlation between *n*_H_ and the association free energy for the empty sites (R^2^ = 0.083). Taken together these results indicate that the Hill coefficient only depends on the Gibbs free energy of interaction (*ΔG* ^o^_int_). This dependence is bi-univocal and, for moderate values of *ΔG* ^o^_int_, it can be approximated by a linear function.

The Hill coefficient is also commonly used to estimate the number of ligand molecules that bind to a receptor and produce a functional effect [25]. However, it has been demonstrated that for systems showing positive cooperativity the Hill coefficient sets the lower limit for the number of interacting binding sites [20,25]. Fig 4 shows that feature but, as it can be observed, all the values between zero and 2 are allowed, in agreement with the idea that the Hill coefficient does not contain reliable information about the total number of binding sites present in a given macromolecule. Experimental studies reinforce this view, for example reported values of the Hill coefficient for binding of oxygen to normal hemoglobin range between 2.8 and 3.4 depending on conditions and type of measurements [14], and upon interaction with the erythrocyte anion transport band 3 this value falls to 1.6 [26].

The minimal value that *n*_H_ can acquire has been also a matter of discussion. The sequential mechanism of Koshland-Némethy-Filmer [27] predicts that at extreme conditions of negative cooperativity *n*_H_ approaches zero. More recently, Abeliovich [18] proposed a novel interpretation of the Hill coefficients based on the statistical behavior of the system, and concluded that the value of the Hill coefficient –experimentally determined at midsaturation- has a lower limit given by the reciprocal of the number of binding sites. Our procedure for calculating the Hill coefficient from the simulated data (i.e. following the same procedures used for experimental data), shows that the Hill coefficient can take values lower than 0.5 for ΔG°_int_ values higher than 5 kJ/mol. This observation is also supported by experimental evidence. When studying the binding of the epidermal growth factor to the extracellular region of the EGF receptor from Drosophila melanogaster (s-dEGFR), Alvarado et al. [28] demonstrate a structural rearrangement, after ligand induced dimerization of s-dEGFR, resulting in negative cooperativity between 2 binding sites. The Hill coefficient determined in that work from a ligand binding experiment is 0.31, a value clearly below the lower limit of 0.5 predicted by Abeliovich.

### Can negative cooperativity and different binding sites be distinguished?

One of the major challenges when studying the interaction between macromolecules and ligands is the distinction between identical sites with negative cooperativity and different classes of sites without interactions. As it was previously mentioned, both binding models exhibit Hill coefficients lower than 1, and thus this coefficient does not provide the desired distinction. The problem can be theoretically formulated considering that the binding isotherms are indistinguishable when their partition functions are identical (see Eq. A12 and A14 in S1 Appendix). Hence, the following relations can be derived:

$$\begin{array}{l} K_{{\text{o}}_{1}}+K_{{\text{o}}_{2}}=2\cdot K_{\text{o}}\\ K_{{\text{o}}_{1}}\cdot K_{{\text{o}}_{2}}=\omega \cdot K_{\text{o}}{}^{2} \end{array}$$ (11)

where *K*_o1_ and *K*_o2_ are the intrinsic equilibrium association constant for site 1 and site 2 (for the two different sites model), and *K*_o_ and ω are the intrinsic equilibrium association constant for the empty sites and the cooperativity factor (for the cooperative binding model).

This system of equations has two solutions:

$$\begin{array}{l} K_{{\text{o}}_{1}}=K_{\text{o}}\cdot \left(1+\sqrt{1-\omega }\right)\\ K_{{\text{o}}_{2}}=K_{\text{o}}\cdot \left(1-\sqrt{1-\omega }\right) \end{array}$$ (12)

There is a trivial root corresponding to the cooperativity factor ω = 1. In this case both roots are equal and correspond to the case of identical and independent sites. On the other hand, when ω > 1, both roots are complex numbers and thus they have no physical meaning. Therefore, the binding isotherm corresponding to a molecular system displaying positive cooperativity cannot be erroneously described by a two sites model. On the contrary, the roots represented by Eq 12 are real when ω < 1. This means that, if Eq 12 holds, the binding isotherms corresponding to a macromolecule with two identical sites displaying negative cooperativity or two different sites without interactions cannot be distinguished.

**Fig 5:**
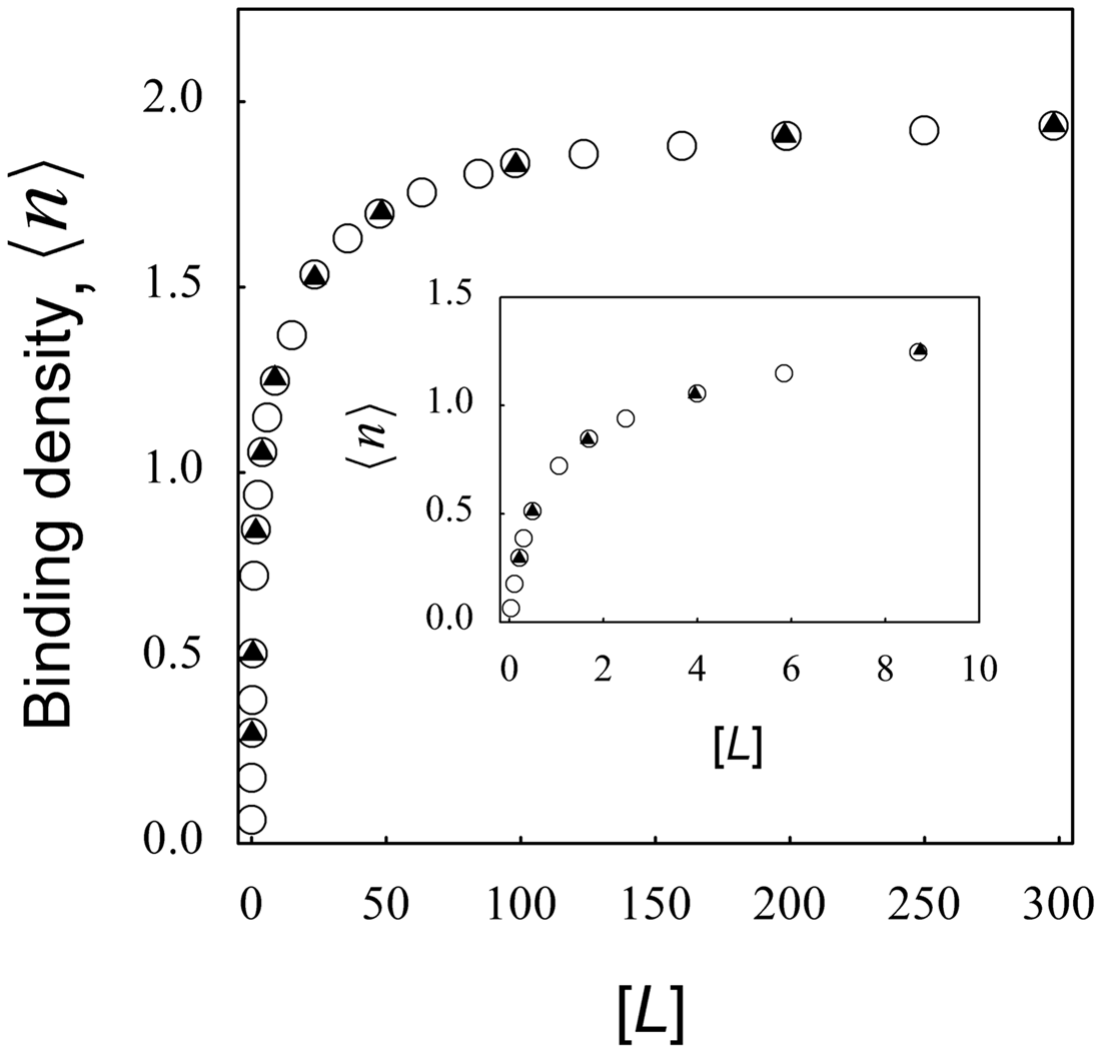
Comparison between negative cooperativity and different sites at equilibrium conditions. Eqs 2–6 were numerically solved using the following kinetic coefficients *k*_1_ = *k*_2_ = 0.55 (μM^-1^s^-1^) and *k*_-1_ = *k*_-2_ = 1 (s^-1^) and ω = 0.33058 for the case of two identical sites for a ligand with negative cooperativity and *k*_1_ = *k*_1(2)_ = 1 (μM^-1^s^-1^); *k*_2_ = *k*_2(1)_ = 0.1 (μM^-1^s^-1^) and *k*_-1_ = *k*_-1(2)_ = *k*_-2_ = *k*_-2(1)_ = 1 (s^-1^) for two classes of binding sites without interactions. Total concentration of macromolecule was in all cases 1 (μM) and the concentrations of ligand were varied from 0.01 to 300 (μM). The average number of occupied sites (〈*n*〉) was calculated using Eq 7 and equilibrium values of 〈*n*〉 and [*L*] were obtained from each curved and plotted as a binding isotherm. The binding isotherms of two identical sites for a ligand with negative cooperativity (black triangles) and two classes of binding sites without interactions (white circles) are shown. Inset shows a zoom of the main plot at low ligand concentrations.

However, the indistinguishability of the thermodynamic equilibrium states does not imply that both systems are identical over the complete time courses of the binding reaction. This idea was previously formulated by Wang & Pan [29], although under two restrictive conditions: 1) irreversible binding, and 2) pseudofirst order conditions. Our modeling approach overcomes these limitations and thus constitutes a valuable tool to explore this hypothesis. For this purpose time course simulations were performed by setting the kinetic coefficients and the cooperativity factor ω, so that Eq 12 were satisfied and binding isotherms were generated for both models (Fig 5). As expected, no differences were found between the equilibrium binding curves, even at very low ligand concentrations (inset to Fig 5), in agreement with the previous thermodynamic analysis.

**Fig 6:**
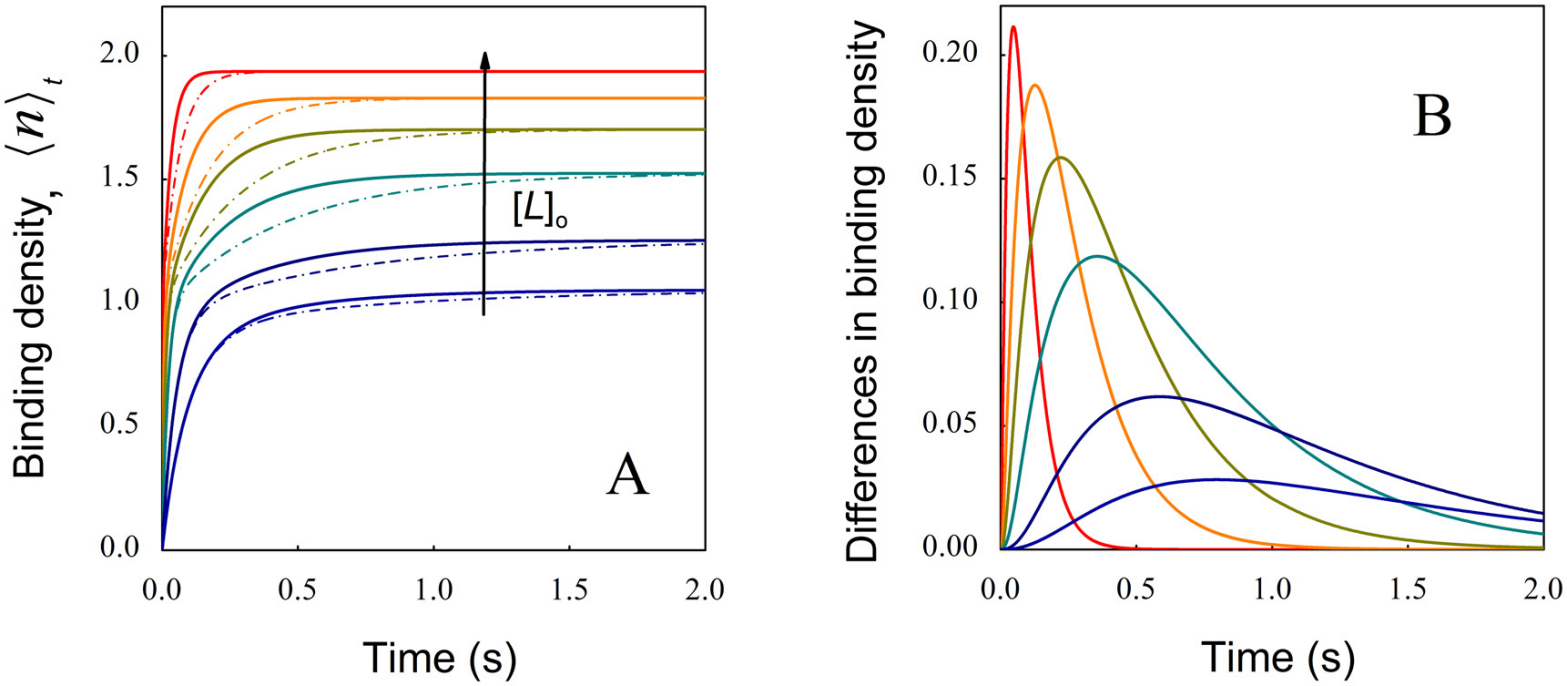
Comparison between negative cooperativity and different sites in pre-equilibrium conditions. (A) Time course of site occupation for a macromolecule with two identical sites for negative cooperativity (continuous lines) and two classes of binding sites without interactions (dash-dotted lines) for the following initial ligand concentrations (μM): 300 (red), 100 (orange), 50 (green), 25 (turquoise), 10 (blue) and 5 (violet). Time courses simulations were obtained under the same conditions as described in Fig 5. (B) The difference Δ〈*n*〉 = 〈*n*〉_coop_—〈*n*〉_diff sites_ was calculated for each ligand concentration and represented as a function of time. For clarity reasons only 6 representative time courses traces that gave rise to the equilibrium data points of Fig 5 are shown.

When comparing the time courses of ligand binding significant differences between both sets of simulated data can be observed (Fig 6A) allowing to identify the corresponding binding model. To highlight this, the difference between the average numbers of occupied sites (Δ〈*n*〉 = 〈*n*〉_coop_−〈*n*〉_diff. sites_) for each total ligand concentration is depicted in Fig 6B. It can be observed that differences, initially small, reach a maximal value at different times (depending on ligand concentration) and become indistinguishable when approaching to equilibrium (Fig 6A and 6B).

Our analysis clearly show that, in contrast to what is observed in equilibrium binding measurements, the reaction time courses for ligand binding to a macromolecule having different classes of sites or negatively interacting sites are not identical. Therefore, we propose a strategy for discrimination between ligand binding models in an experimental context that can be summarized as follows:

1. *Experimental stage*.
  - The macroscopic equilibrium constants are calculated from the ligand binding isotherms [30].
  - If the equilibrium analysis indicates that such isotherms can be described by a model of identical sites with negative cooperativity or different sites without interactions, time course experiments following ligand binding to the macromolecule will be carried out to distinguish between both models [31,32,33].
2. *Data analysis stage*.
  - Both ligand binding kinetic models must be fitted by non linear regression to the set of experimental time courses [21,24,34]. Importantly, the rate constants of each model should be constrained to satisfy the relations given by the macroscopic equilibrium constants obtained in step 1.
  - Finally, by using appropriate statistical criteria, the experimenter should select the model that better describe the experimental data.

It is important to notice that for some values of the kinetic coefficients, the possibility of relaxing the conditions imposed by Eq 12 in the fitting procedure, make the global differences between models small enough so that they are in the order of the experimental error. Indeed, this is not the general case. In fact, we have previously reported an experimental study on the interaction between the small ligand 1-anilino-naphthalene-8-sulfonate and bovine serum albumin, showing the efficacy of the kinetic approach to identify a two classes of sites model for that system [21].

### Hidden sites in binding systems with negative cooperativity

**Fig 7:**
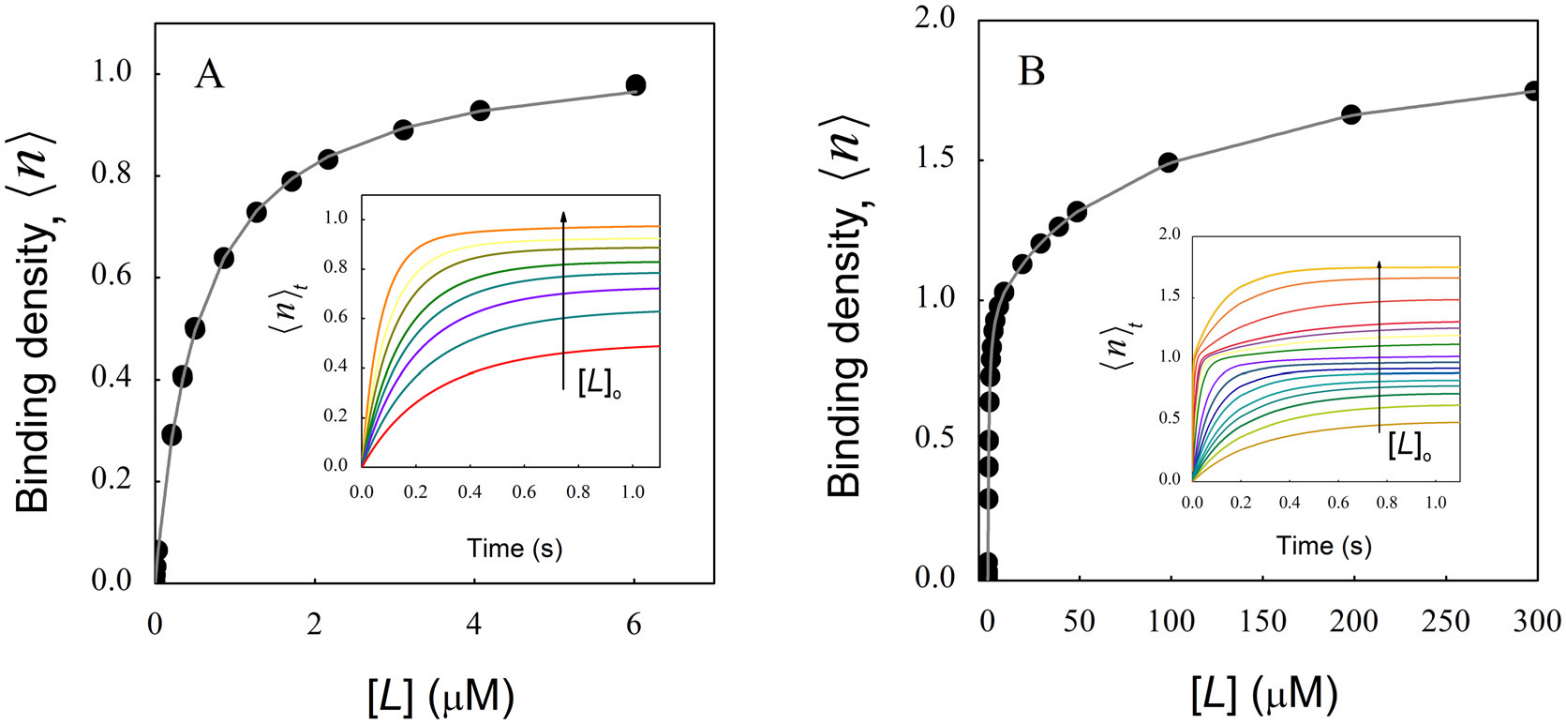
Systems with strong negative cooperativity can mask binding sites. Biding isotherms obtained from kinetic simulations of a macromolecule with two binding sites and high negative cooperativity (ω = 0.02 and *K*_o_ = 1 (μM^-1^)) for ligand concentrations up to 6 μM (A) or 300 mM (B). Insets show the simulated time courses used to generate the binding isotherms shown in both panels. Arrows indicate increasing ligand concentration. Notice the change in the kinetic behavior for high ligand concentrations in panel B.

It is well known that sites in macromolecules having very low affinity for ligands may not be experimentally detected, and a similar problem could be envisaged for systems exhibiting strong negative cooperativity. To explore this hypothesis, we performed simulations corresponding to very low values of the cooperativity factor ω. Fig 7A shows the binding isotherm corresponding to a macromolecule with two identical sites and strong negative cooperativity (ω = 0.02 and *K*_o_ = 1 (μM^-1^)) for ligand concentrations up to 6 (μM). It can be observed that the binding isotherm saturates at a binding density near one. Continuous line shows the fit of a simple hyperbola which described satisfactorily the behavior of the simulated data at equilibrium, with best-fit parameter values 〈*n*〉 = 1.055 ± 0.002 and *K*_o_ = 1.78 ± 0.02 (μM^-1^). As expected, calculation of the Hill coefficient with this set of data gives a value of 1. These results show that an experimental study of ligand binding to a macromolecule with two identical sites and with strong negative cooperativity, would indicate that the macromolecule has a unique binding site with a ligand affinity two times higher than the real affinity. This higher apparent affinity is due to a “statistical effect” given by the fact that a ligand molecule has two possibilities for binding to the macromolecule instead of one as it would happen if the molecule had a unique site. This is also evident when comparing the partition function given by Eq. A14 (S1 Appendix) with that corresponding to the binding at a unique site (Eq. C3 in S3 Appendix). When ω is very low, the last term in Eq. A14 becomes negligible and this equation becomes similar to Eq. C3 despite a factor 2 affecting the association constant. In this scenario, the system at the apparent saturating conditions will be composed by macromolecules loaded in average with one ligand, but about half of them will have the ligand bound to site 1 and the other half at site 2. It is worth noting that there is no indication in the results shown in Fig 7A suggesting that exploration of higher ligand concentrations is needed, since the binding isotherm appear to have reach saturation. Fig 7B shows the binding isotherm for the same macromolecule but now including much higher ligand concentrations. In this plot the existence of a second site becomes clear. Nevertheless, in true experimental conditions, increasing ligand concentration might be challenging due to practical limitations such as ligand solubility or problems with signal detection. Furthermore, it is important to note that the values of ligand concentration required for detecting the “hidden sites” depend on the value of the association constant for the empty sites (*K*_o_) and the cooperativity factor ω, being these concentrations higher for lower *K*_o_ and ω values. A kinetic experiment could be envisaged to highlight the existence of the hidden site. However the time courses corresponding to the lower ligand concentrations (inset to Fig 7A) follow monoexponential curves, a typical feature of binding to a unique site under pseudo-first order conditions (see S3 Appendix). It can be observed that a more complex kinetic behavior is evident only at high ligand concentrations (inset to Fig 7B), when the equilibrium binding isotherm also indicates the existence of the second site.

## Conclusion

In this work we show that simulation of phenomenological models constitutes a powerful tool to explore binding processes. This procedure allows to generate equilibrium binding isotherms from the time courses of the binding reaction and, from these data, the Hill coefficients can be determined. We show that Hill coefficients estimated from these isotherms have a bi-univocal relation with the Gibbs free energy of interaction among binding sites and their values are independent of the free energy of ligand association to the empty sites. Furthermore, a careful exploration of the simulated data shows distinctive features between the binding time courses corresponding to negative cooperativity and different classes of binding sites, although they are undistinguishable at equilibrium. In this way, our results also highlight the usefulness of pre-equilibrium time-resolved strategies to explore binding models as a key complement of equilibrium studies. Additionally, simulated results show that under conditions of strong negative cooperativity, the existence of some binding sites can be overlooked. We show that experiments at very high ligand concentrations (when compatible with solubility and stability conditions) are a valuable tool to unmask such sites.

## Supporting Information

S1 Appendix Binding models and isotherms. (PDF)

S2 Appendix Calculation of Hill coefficients. (PDF)

S3 Appendix Ligand binding to a single site in a macromolecule. (PDF)

## References

[1] Garbett NC, Chaires JB. Binding: a polemic and rough guide. Methods Cell Biol. 2008; 84: 3–23. 17964926 10.1016/S0091-679X(07)84001-5

[2] Gutfreund H. Kinetics for the life sciences: Receptors, transmitters and catalysts. Cambridge: Cambridge University Press; 1995.

[3] Hager GL, McNally JG, Misteli T. Transcription dynamics. Mol Cell. 2009; 35: 741–753. 10.1016/j.molcel.2009.09.005 19782025 PMC6326382

[4] Freiburger L, Miletti T, Zhu S, Baettig O, Berghuis A, Auclair K, et al Substrate-dependent switching of the allosteric binding mechanism of a dimeric enzyme. Nat Chem Biol. 2014; 10: 937–942. 10.1038/nchembio.1626 25218742

[5] Dodes Traian MM, Cattoni DI, Levi V, González Flecha FL. A two-stage model for lipid modulation of the activity of integral membrane proteins. PLoS one 2010; 7: e39255. 10.1371/journal.pone.0039255 PMC3378530 22723977

[6] Hillier BJ, Rodriguez HM, Gregoret LM. Coupling protein stability and protein function in *Escherichia coli* CspA. Fold Des. 1998; 3: 87–93. 9565753 10.1016/S1359-0278(98)00014-5

[7] Levi V, Rossi JPFC, Castello PR, González Flecha FL. Structural significance of the plasma membrane calcium pump oligomerization. Biophys J. 2002; 82: 437–446. 11751330 10.1016/s0006-3495(02)75408-8 PMC1302483

[8] Shcheynikov N, Son A, Hong JH, Yamazaki O, Ohana E, Kurtz I, et al Intracellular Cl^-^ as a signaling ion that potently regulates Na^+^/HCO3- transporters. Proc Natl Acad Sci U S A. 2015A 112: E329–337. 25561556 10.1073/pnas.1415673112 PMC4311818

[9] Ruscio JZ, Kumar D, Shukla M, Prisant MG, Murali TM, Onufriev AV, et al Atomic level computational identification of ligand migration pathways between solvent and binding site in myoglobin. Proc Natl Acad Sci U S A. 2008; 105: 9204–9209. 10.1073/pnas.0710825105 18599444 PMC2453746

[10] Sakaguchi M, Shingo T, Shimazaki T, Okano HJ, Shiwa M, Ishibashi S, et al A carbohydrate-binding protein, Galectin-1, promotes proliferation of adult neural stem cells. Proc Natl Acad Sci U S A. 2006; 103: 7112–7117. 16636291 10.1073/pnas.0508793103 PMC1447526

[11] Edelstein SJ, Le Novere N. Cooperativity of allosteric receptors. J Mol Biol. 2013; 425: 1424–1432. 10.1016/j.jmb.2013.03.011 23523898

[12] Barcroft J, Hill AV. The nature of oxyhæmoglobin, with a note on its molecular weight. J Physiol. 1910; 39: 411–428. 16992995 10.1113/jphysiol.1910.sp001350 PMC1533721

[13] Hill AV. The possible effects of the aggregation of the molecules of haemoglobin on its dissociation curves. J Physiol. 1910; 40: iv–vii.

[14] Holt JM, Ackers GK. The Hill coefficient. Inadequate resolution of cooperativity in human hemoglobin. Methods Enzymol. 2009; 455: 193–212. 10.1016/S0076-6879(08)04207-9 19289207

[15] Adair GS, Bock AV, Field JH. The hemoglobin system. VI. The oxygen dissociation curve of hemoglobin. J Biol Chem. 1925; 63: 529–545.

[16] Weber G. Protein interactions. New York: Chapman and Hall; 1992.

[17] Ruzicka FJ, Frey PA. Kinetic and spectroscopic evidence of negative cooperativity in the action of lysine 2,3-aminomutase. J Phys Chem B. 2010; 114: 16118–16124. 10.1021/jp103856m 20608698 PMC4337230

[18] Abeliovich H. An empirical extremum principle for the Hill coefficient in ligand-protein interactions showing negative cooperativity. Biophys J. 2005 89: 76–79. 15834004 10.1529/biophysj.105.060194 PMC1366580

[19] Koshland DE, Hamadani K. Proteomics and models for enzyme cooperativity. J Biol Chem. 2002; 277: 46841–46844. 12189158 10.1074/jbc.R200014200

[20] Weber G, Anderson SR. Multiplicity of binding. range of validity and practical test of Adair's equation. Biochemistry. 1965; 4: 1942–1947.

[21] Cattoni DI, Kaufman SB, González Flecha FL. Kinetics and thermodynamics of the interaction of 1-anilino-naphthalene-8-sulfonate with proteins. Biochim Biophys Acta. 2009; 1794: 1700–1708. 10.1016/j.bbapap.2009.08.007 19683079

[22] Klotz IM. Protein interactions with small molecules. Acc Chem Res. 1974; 7: 162–168.

[23] Wyman J. Linked functions and reciprocal effects in hemoglobin: A second look. Adv Prot Chem. 1964; 19: 223–286. 10.1016/s0065-3233(08)60190-4 14268785

[24] Kemmer G, Keller S. Nonlinear least-squares data fitting in Excel spreadsheets. Nat Protoc. 2010; 5: 267–281. 10.1038/nprot.2009.182 20134427

[25] Weiss JN. The Hill equation revisited: Uses and misuses. FASEB J. 1997; 11: 835–841. 9285481

[26] Walder JA, Chatterjee R, Steck TL, Low PS, Musso GF, Kaiser ET, et al The interaction of hemoglobin with the cytoplasmic domain of band 3 of the human erythrocyte membrane. J Biol Chem. 1984; 259: 10238–10246. 6469962

[27] Koshland DE, Némethy G, Filmer D. Comparison of experimental binding data and theoretical models in proteins containing subunits. Biochemistry. 1966; 5: 365–385. 5938952 10.1021/bi00865a047

[28] Alvarado D, Klein DE, Lemmon MA. Structural basis for negative cooperativity in growth factor binding to an EGF receptor. Cell. 2010; 142: 568–579. 10.1016/j.cell.2010.07.015 20723758 PMC2925043

[29] Wang Z-X, Pan X-M. Kinetic differentiation between ligand-induced and pre-existent asymmetric models. FEBS Letters. 1996 388: 73–75. 8654593 10.1016/0014-5793(96)00498-x

[30] Bujalowski W, Jezewska MJ. Quantitative thermodynamic analyses of spectroscopic titration curves. J Mol Struct. 2014; 1077: 40–50. 25284889 10.1016/j.molstruc.2014.04.041 PMC4181404

[31] Hargrove MS. Ligand binding with stopped-flow rapid mixing. Methods Mol Biol. 2005; 305: 323–342. 15940005 10.1385/1-59259-912-5:323

[32] Pollard TD, De La Cruz EM. Take advantage of time in your experiments: a guide to simple, informative kinetics assays. Mol Biol Cell. 2013; 24: 1103–1110. 10.1091/mbc.E13-01-0030 23580192 PMC3623632

[33] As the kinetic analysis of these curves is performed using specific software that numerically solve the differential equations associated with each ligand binding model, it will not be necessary to carry out those experiments under pseudo-first order conditions

[34] Levi V, Villamil Giraldo AM, Castello PR, Rossi JP, González Flecha FL. Effects of phosphatidylethanolamine glycation on lipid-protein interactions and membrane protein thermal stability. Biochem J. 2008; 416: 145–152. 10.1042/BJ20080618 18564061

