# Supplementary material for: Cooperativity in Binding Processes: New Insights from Phenomenological Modeling
Source: PLoS One. 2015 Dec 30;10(12):e0146043. doi: 10.1371/journal.pone.0146043 (PMC4696654; doi:10.1371/journal.pone.0146043)
Supplement: S1 Appendix — (PDF) [file pone.0146043.s001.pdf]

Supplementary Information

Cooperativity in binding processes:
New insights from phenomenological modeling

Diego I. Cattoni[1,2], Osvaldo Chara[3,4] Sergio B. Kaufman[1] and F. Luis González Flecha[1]

[1] Laboratorio de Biofísica Molecular, Instituto de Química y Fisicoquímica Biológicas. Universidad de Buenos Aires – CONICET, Argentina. [2] Centre de Biochimie Structurale, Université de Montpellier 1 and 2, France. [3] Instituto de Fisica de Líquidos y Sistemas Biológicos, Universidad Nacional de La Plata - CONICET, Argentina. [4] Center for Information Services and High Performance Computing, Technische Universität Dresden, Germany.

Appendix A: Binding models and isotherms

Binding models. The binding parameters accessible from equilibrium titration experiments are the binding stoichiometry and the macroscopic association constants ($\mathbf{K_i}$), which govern the sequential binding of one, two or more ligands to the macromolecule. The relationship between macroscopic constants and the microscopic states for a binding reaction between a macromolecule and two ligands is given by:

$$M + L \rightleftharpoons LM \qquad \mathbf{K}_1 = \frac{[LM]}{[L]\cdot[M]} = \frac{\left[L_1M^{1,0}\right]+\left[L_1M^{0,1}\right]}{[L]\cdot[M]} \tag{A1}$$

$$LM + L \rightleftharpoons L_2M \qquad \mathbf{K}_2 = \frac{[L_2M]}{[L]\cdot[L_1M]} = \frac{\left[L_2M^{1,1}\right]}{[L]\cdot\left(\left[L_1M^{1,0}\right]+\left[L_1M^{0,1}\right]\right)} \tag{A2}$$

Macroscopic equilibrium constants contain -hidden inside them- intrinsic association constants, statistical, and eventually cooperativity factors. Binding models can be distinguished according to the relationship between the magnitudes of the macroscopic equilibrium constants. The explicit relation between macroscopic ($\mathbf{K_i}$) and microscopic (K_{o_i}), association constants for the binding reaction depicted in Fig. 2 are:

$$\mathbf{K}_1 = K_{o_1} + K_{o_2} \tag{A3}$$

$$\mathbf{K}_2 = \frac{\omega \cdot K_{o_1} \cdot K_{o_2}}{K_{o_1} + K_{o_2}} \tag{A4}$$

where ω is a factor that takes a value equal to one for independent binding sites and any other real positive value for non-independent binding sites.

The simplest case of identical and independent sites is observed when all the association and dissociation kinetic coefficients depicted in Fig. 2 (main article) have identical values. Thus, the intrinsic equilibrium association constant (K_o) for each site can be defined as:

$$K_o = \frac{k_1}{k_{-1}} = \frac{k_{2(1)}}{k_{-2(1)}} = \frac{k_2}{k_{-2}} = \frac{k_{1(2)}}{k_{-1(2)}} = K^{\dagger} \cdot e^{-\frac{\Delta G^o_{assoc}}{R \cdot T}} \quad \text{(A5)}$$

where ΔG^{o}_{assoc} the Gibbs free energy change for the association between L and M each of them at the thermodynamic reference state and $K^{\dagger} \equiv 1\ M^{-1}$.

When the macromolecule has two classes of independent binding sites, one of the sites may have an intrinsic association constant (K_{o_2}) δ-times that of the other site (K_{o_1}), then:

$$K_{o_1} = \frac{k_1}{k_{-1}} = \frac{k_{1(2)}}{k_{-1(2)}} = K^{\dagger} \cdot e^{-\frac{\Delta G^o_{1\,assoc}}{R \cdot T}} \qquad K_{o_2} = \frac{k_2}{k_{-2}} = \frac{k_{2(1)}}{k_{-2(1)}} = \delta \frac{k_1}{k_{-1}} = K^{\dagger} \cdot e^{-\frac{\Delta G^o_{2\,assoc}}{R \cdot T}} \quad \text{(A6)}$$

and the difference between the association Gibbs free energies for the two sites is given by:

$$\Delta\Delta G^o = \Delta G^o_{2\,assoc} - \Delta G^o_{1\,assoc} = -R \cdot T \cdot \ln \frac{K_{o_2}}{K_{o_1}} = -R \cdot T \cdot \ln \delta \quad \text{(A7)}$$

Finally, if the molecule has two interacting binding sites cooperativity can be observed. If the intrinsic association constants for both empty sites are identical but the binding of ligand at one site modifies the association constant of the other site by a factor ω, then we are in the presence of positive (ω > 1) or negative (ω < 1) cooperativity. The intrinsic association constants for the binding of the first (K_o) and second (K_o') ligand molecule are now represented by:

$$K_o = \frac{k_1}{k_{-1}} = \frac{k_2}{k_{-2}} = K^{\dagger} \cdot e^{-\frac{\Delta G^o_{1\,assoc}}{R \cdot T}} \qquad K_o' = \frac{k_{2(1)}}{k_{-2(1)}} = \frac{k_{1(2)}}{k_{-1(2)}} = \omega \frac{k_1}{k_{-1}} = K^{\dagger} \cdot e^{-\frac{\Delta G^o_{2\,assoc}}{R \cdot T}} \quad \text{(A8)}$$

As defined by Wyman [1], the free energy of interaction between sites will be given by:

$$\Delta G^o_{\text{int}} = \Delta G^o_{2\,assoc} - \Delta G^o_{1\,assoc} = -R \cdot T \cdot \ln \frac{K_{o_2}}{K_{o_1}} = -R \cdot T \cdot \ln \omega \quad \text{(A9)}$$

Binding isotherms. The binding isotherms are the graphical representation of the average number of bound ligand molecules per macromolecule (⟨n⟩, also referred as binding density) as a function of the free ligand concentration at a given temperature.

Binding isotherms are usually obtained by measuring a signal (typically spectroscopic or calorimetric) reporting on the binding density at different ligand-macromolecule mole ratios.

The mathematical expression for the binding isotherm corresponding to a given binding model can be obtained from the corresponding grand canonical molecular partition function formulated in terms of the ligand concentration [1,2,3].

For the binding of a ligand to a macromolecule with two identical sites without interactions the partition function is defined as:

$$\Xi_L = 1 + 2 \cdot K_o \cdot [L] + K_o^{\,2} \cdot [L]^2 \quad \text{(A10)}$$

and its derivate is the binding density ⟨n⟩

$$\frac{\partial \ln \Xi_L}{\partial \ln [L]} = \frac{2 \cdot K_o \cdot [L]}{1 + K_o \cdot [L]} = \langle n \rangle \quad \text{(A11)}$$

For the binding of two ligand molecules at different sites without interactions, the partition function is:

$$\Xi_L = 1 + K_{o_1} \cdot [L] + K_{o_2} \cdot [L] + K_{o_1} \cdot K_{o_2} [L]^2 \quad \text{(A12)}$$

and the corresponding binding density is given by:

$$\langle n \rangle = \frac{K_{o_1} \cdot [L]}{1 + K_{o_1} \cdot [L]} + \frac{K_{o_2} \cdot [L]}{1 + K_{o_2} \cdot [L]} \quad \text{(A13)}$$

Finally, when the binding of a ligand to a first site affects the affinity of the second site by a factor ω, we are in the presence of interacting sites where the partition function is now:

$$\Xi_L = 1 + 2 \cdot K_o \cdot [L] + \omega \cdot K_o^2 \cdot [L]^2 \quad \text{(A14)}$$

and the binding density will be given by:

$$\langle n \rangle = \frac{2 \cdot K_o \cdot [L] + 2 \cdot \omega \cdot K_o^2 \cdot [L]^2}{1 + K_o \cdot [L] + \omega \cdot K_o^2 \cdot [L]^2} \quad \text{(A15)}$$

where K_{o1} represents the intrinsic association constant for the empty sites.

References

1. Wyman J (1964) Linked functions and reciprocal effects in hemoglobin: A second look. Adv Prot Chem 19: 223-286.
2. Wyman J, Gill, S.J. (1990) Binding and linkage. The functional chemistry of biological macromolecules. Mill Valley, California University Science Books.
3. Hill TL (1985) Cooperativity theory in biochemistry: Steady-state and equilibrium systems: Springer.
