# Supplementary material for: Cooperativity in Binding Processes: New Insights from Phenomenological Modeling
Source: PLoS One. 2015 Dec 30;10(12):e0146043. doi: 10.1371/journal.pone.0146043 (PMC4696654; doi:10.1371/journal.pone.0146043)
Supplement: S2 Appendix — (PDF) [file pone.0146043.s002.pdf]

Supplementary Information

Cooperativity in binding processes: New insights from phenomenological modeling

Diego I. Cattoni[1,2], Osvaldo Chara[3,4] Sergio B. Kaufman[1]and F. Luis González Flecha[1]

[1] Laboratorio de Biofísica Molecular, Instituto de Química y Fisicoquímica Biológicas. Universidad de Buenos Aires – CONICET, Argentina. [2] Centre de Biochimie Structurale, Université de Montpellier 1 and 2, France. [3] Instituto de Fisica de Líquidos y Sistemas Biológicos, Universidad Nacional de La Plata - CONICET, Argentina. [4] Center for Information Services and High Performance Computing, Technische Universität Dresden, Germany.

Appendix B: Calculation of Hill coefficients.

Cooperative binding can be explored by analyzing binding isotherms in terms of the Wyman´s Hill plot [1].

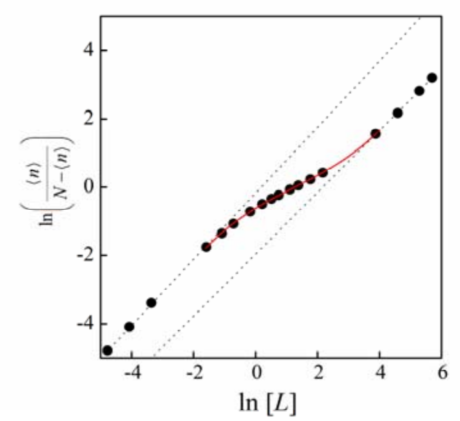

Figure B1. Wyman´s Hill plot for ligand binding to two identical sites exhibiting negative cooperativity Binding isotherms were generated as described in Figure 3 for ω = 0.10 (ΔG^{o}_{int} = 5.71 kJ/mol) , and re-plotted according to the procedure proposed by Wyman [1]. The continuous red line represents a third order polynomial fitted to the data in the transition region with the best fitting parameters a_3 = 0.0202; a_2= 0.0761 a_1= 0.5537 and a_0= 0.6193. The Hill coefficient for this representative example was n_H = 0.458.

This plot has two asymptotic regions at low and high free ligand concentration, being the slope of the asymptotes equal to unity. Hill coefficients are usually calculated as the slope of a linear function fitted to the data in the transition region corresponding to half saturation of the sites.

In this work we fit a third order polynomial function.

$$y = a_3 \cdot x^3 + a_2 \cdot x^2 + a_1 \cdot x + a_0 \qquad \text{(B1)}$$

Thus, the Hill coefficient is calculated as the maximum (or minimum) value of the first derivative of B1 in the region linking the two asymptotic regions

By taking the first derivative

$$\frac{dy}{dx} = 3 \cdot a_3 \cdot x^2 + 2 \cdot a_2 \cdot x + a_1 \qquad \text{(B2)}$$

This derivative has a maximum (for positive cooperativity) or a minimum (for negative cooperativity) corresponding to the inflection point of Eq. B1.

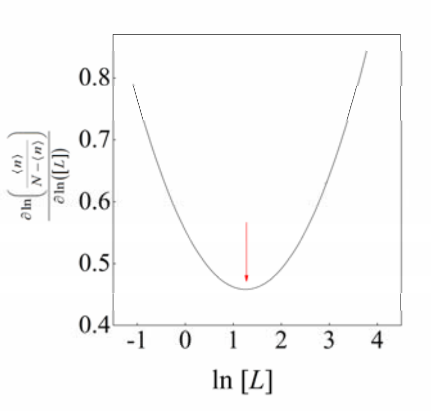

Figure B2. Equation B2 is represented with the best fitted parameter values obtained in Figure B1. The minimal value of this derivative (indicated by the arrow) corresponds to the Hill coefficient.

The coordinates of the inflection point is obtained by equaling the second derivative to zero

$$\frac{d^2y}{dx^2} = 6 \cdot a_3 \cdot x + 2 \cdot a_2 = 0 \qquad \text{(B3)}$$

Thus

$$x = \frac{2 \cdot a_2}{6 \cdot a_3}. \qquad \text{(B4)}$$

Replacing this value of *x* in Eq. B2 an accurate Hill coefficient value can be estimated.

$$n_{\mathrm{H}} = a_1 - \frac{a_2^{\,2}}{3 \cdot a_3} \qquad \text{(B5)}$$

References

1. Wyman J (1964) Linked Functions and Reciprocal Effects in Hemoglobin: A Second Look. Adv Prot Chem 19: 223-286.
