# Supplementary material for: Cooperativity in Binding Processes: New Insights from Phenomenological Modeling
Source: PLoS One. 2015 Dec 30;10(12):e0146043. doi: 10.1371/journal.pone.0146043 (PMC4696654; doi:10.1371/journal.pone.0146043)
Supplement: S3 Appendix — (PDF) [file pone.0146043.s003.pdf]

Supplementary Information

Cooperativity in binding processes: New insights from phenomenological modeling

Diego I. Cattoni[1,2], Osvaldo Chara[3,4] Sergio B. Kaufman[1]and F. Luis González Flecha[1]

[1] Laboratorio de Biofísica Molecular, Instituto de Química y Fisicoquímica Biológicas. Universidad de Buenos Aires – CONICET, Argentina. [2] Centre de Biochimie Structurale, Université de Montpellier 1 and 2, France. [3] Instituto de Fisica de Líquidos y Sistemas Biológicos, Universidad Nacional de La Plata - CONICET, Argentina. [4] Center for Information Services and High Performance Computing, Technische Universität Dresden, Germany.

Appendix C: Ligand binding to a single site in a macromolecule.

The binding of a ligand L to a macromolecule M with a single site can be described by:

$$M + L \underset{k_{-1}}{\overset{k_1}{\rightleftharpoons}} LM \tag{C1}$$

With an equilibrium constant

$$K_o = \frac{k_1}{k_{-1}} = K^{\dagger} \cdot e^{-\frac{\Delta G^{o}_{assoc}}{R \cdot T}} \tag{C2}$$

The partition function for this scheme will be [1,2]:

$$\Xi_L = 1 + K_o \cdot [L] \tag{C3}$$

and the corresponding binding density:

$$\langle n \rangle \equiv \frac{[ML]}{[M]_0} = \frac{K_o \cdot [L]}{1 + K_o \cdot [L]} \tag{C4}$$

The time course of the binding density will be given by:

$$\frac{d\langle n \rangle}{dt} = \frac{1}{[M]_0} \cdot \frac{d[ML]}{dt} = \frac{1}{[M]_0} \cdot \left(k_1 \cdot [L] \cdot [M] - k_{-1} \cdot [LM] \right) \tag{C5}$$

Rearranging and integrating over time between an initial condition, where only M and L are present (with $[L] >> [M]$), and the equilibrium final state we obtain:

$$\langle n \rangle_t = \frac{K_{o_1} \cdot [L]}{1 + K_{o_1} \cdot [L]} \cdot \left(1 - e^{-(k_1[L] + k_{-1}) \cdot t} \right) \tag{C6}$$

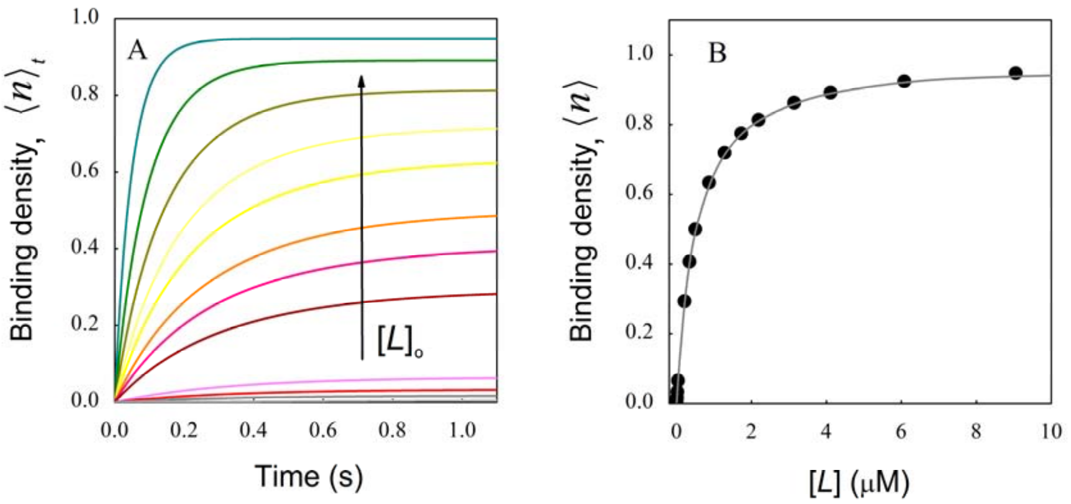

Figure C1. Site occupation for a macromolecule with one site for a ligand. (A) Equations 16-20 (main text) were numerically solved with k_1 = 2 μM^{-1}s^{-1} and k_{-1} = 1 s^{-1} and a value of zero for all the other kinetic coefficients. Total concentration of macromolecule was 1 μM and the concentration of ligand was varied from 0.01 to 300 μM. Arrow indicates increasing ligand concentrations. (B) The equilibrium values of ⟨*n*⟩ and [*L*] were obtained from each curve and plotted as a binding isotherm.

References

1. Wyman J, Gill, S.J. (1990) Binding and Linkage. The Functional Chemistry of Biological Macromolecules. Mill Valley, California University Science Books.

2. Hill TL (1985) Cooperativity Theory in Biochemistry: Steady-State and Equilibrium Systems: Springer.
